# Cardiac cGMP/cGMP-dependent protein kinase I signalling requires cysteine-rich LIM-only protein 4 (CRP4) to oppose angiotensin II induced hypertrophy and fibrosis

**DOI:** 10.1186/2050-6511-14-S1-P68

**Published:** 2013-08-29

**Authors:** Julia Straubinger, Peter Ruth, Robert Lukowski

**Affiliations:** 1 Pharmakologie, Toxikologie und Klinische Pharmazie, Institut für Pharmazie, Universität Tübingen, Tübingen, Germany

## Background

Cardiac hypertrophy is an adaptive response of the heart to many cardio-vascular disorders including hypertension, infarction and defects of the valves. Elevated levels of cardiac cyclic guanosine-3',5'-monophosphate (cGMP) activate cGMP-dependent protein kinase I (cGKI), which reportedly exhibited either anti-fibrotic and/or anti-hypertrophic effects or did not change the cardiac remodeling responses [1-5]. Based on these findings, we and others suggested that the ability of natriuretic peptides (NP) to oppose detrimental changes via cGMP/cGKI might strongly depend on the growth-promoting neuro-hormonal signals and stresses. Aiming to dissect the molecular details underlying cardiac cGMP signaling, we investigated the cysteine-rich LIM-only protein 4 (CRP4) as a novel target of cardio-vascular cGMP *in vivo*. CRP4 is phosphorylated at Ser-104 by cGMP/cGKI [6-8] and a highly related homologue of the muscle LIM protein CRP3/MLP, which has been linked to dilated and hypertrophic cardiomyopathies in mice and humans [9,10].

## Materials and methods

A patho-/physiological growth adaption of the heart muscle was induced either by an increase in afterload upon chronic angiotensin II (AngII) infusions (2 mg/kg/d) or healthy exercise training using a duration-controlled swimming protocol in CRP4 knockout (KO), wild type (WT) and heterozygous (HET) littermates. The extent of the cardiac growth response was defined by referring changes in heart weight (HW) to body weight (HW/BW) and tibia length (HW/TL). Hypertrophic marker genes, putative effects of AngII on components of the NP/cGMP/cGKI pathway and the expression pattern of other members of the CRP protein family were analyzed in total mRNA and protein preparations isolated from healthy and hypertrophic ventricles. These experiments were corroborated by the localization of CRP4 in the myocardium and Sirius Red staining as a quantitative measure of fibrosis.

## Results

CRP4 mRNA and protein levels were significantly reduced in HET hearts and absent from KO muscles. HW/BW and HW/TL ratios of all three genotypes did not differ at baseline, however, cardiomyocyte size and heart ratios were elevated in CRP4 HET and KO animals in response to the AngII infusions. Interstitial fibrosis was significantly stimulated by AngII in CRP4-deficient and HET hearts, whereas the production of anti-fibrotic factors such as BNP was diminished. Importantly, no differences between the genotypes in cardiac mass or the amount of fibrosis were detected upon swimming exercises.

## Conclusion

The increased susceptibility of CRP4-deficient hearts to chronic AngII exposure indicates that beneficial effects of cGMP/cGKI to oppose Gαq-mediated signaling require cardiac CRP4.
